# Role of microRNA221 in regulating normal mammary epithelial hierarchy and breast cancer stem-like cells

**DOI:** 10.18632/oncotarget.2888

**Published:** 2015-02-17

**Authors:** Jia Ke, Zhiju Zhao, Su-Hyung Hong, Shoumin Bai, Zhen He, Fayaz Malik, Jiahui Xu, Lei Zhou, Weilong Chen, Rachel Martin-Trevino, Xiaojian Wu, Ping Lan, Yongju Yi, Christophe Ginestier, Ingrid Ibarra, Li Shang, Sean McDermott, Tahra Luther, Shawn G. Clouthier, Max S. Wicha, Suling Liu

**Affiliations:** ^1^ Department of Colorectal Surgery, Sixth Affiliated Hospital of Sun Yat-Sen University, Guangzhou, China; ^2^ Innovation Center for Cell Biology and The CAS Key Laboratory of Innate Immunity and Chronic Disease, School of Life Sciences and Medical Center, University of Science & Technology of China, Hefei, Anhui, China; ^3^ Department of Oral Microbiology, School of Dentistry Kyungpook National University, Jung-gu, Daegu, South Korea; ^4^ Department of Oncology, Sun Yat-Sen Memorial Hospital, Sun-Yat-Sen University, Guangzhou, China; ^5^ Comprehensive Cancer Center, Department of Internal Medicine, University of Michigan, Ann Arbor, MI, USA; ^6^ Network Information Center, Sixth Affiliated Hospital of Sun Yat-Sen University, Guangzhou, China; ^7^ Centre de Recherche en Cancérologie de Marseille, Laboratoire d'Oncologie Moléculaire, UMR891 Inserm/Institut Paoli-Calmettes, Université de la Méditerranée, Marseille, France; ^8^ Cold Spring Harbor Laboratory, Program in Genetics and Bioinformatics, Cold Spring Harbor, NY, USA

**Keywords:** miR-221, breast stem-like cells, differentiation, hierarchy

## Abstract

Increasing evidence suggests that lineage specific subpopulations and stem-like cells exist in normal and malignant breast tissues. Epigenetic mechanisms maintaining this hierarchical homeostasis remain to be investigated. In this study, we found the level of microRNA221 (miR-221) was higher in stem-like and myoepithelial cells than in luminal cells isolated from normal and malignant breast tissue. In normal breast cells, over-expression of miR-221 generated more myoepithelial cells whereas knock-down of miR-221 increased luminal cells. Over-expression of miR-221 stimulated stem-like cells in luminal type of cancer and the miR-221 level was correlated with clinical outcome in breast cancer patients. Epithelial-mesenchymal transition (EMT) was induced by overexpression of miR-221 in normal and breast cancer cells. The EMT related gene ATXN1 was found to be a miR-221 target gene regulating breast cell hierarchy. In conclusion, we propose that miR-221 contributes to lineage homeostasis of normal and malignant breast epithelium.

## INTRODUCTION

Mammary stem cells (MaSCs) are defined in part by the ability to self-renew and differentiate. They initially generate uncommitted progenitors and give rise to mature luminal cells with secretory functions and basal/myoepithelial cells with contractile functions. Corresponding to the lineages of normal breast tissue, human breast cancers can be classified into the subtypes as luminal, basal, Her2^+^, and Claudin-low by transcriptional profiling. These subtypes exhibit critical differences in patient survival and response to treatment [1]. Profiling of cell subpopulations purified from normal and malignant breast tissue revealed that, the mRNA signature of normal luminal progenitor cells is most similar to the basal type of breast cancer, whereas the MaSCs signature most closely resembles Claudin-low type of breast cancer [2]. Furthermore, using a human-in-mouse *in vivo* transformation model, Keller et al showed that carcinogenic mutations in mature luminal cells induced luminal type of cancer and mutations in myoepithelial-like cells gave rise to Claudin-low tumor [3]. The connections between normal and malignant hierarchies suggest a similar regulatory mechanism, which require further investigation.

MicroRNAs (miRNAs), one of noncoding RNAs containing approximately 22 nt in length, downregulate expression of hundreds of genes simultaneously, and may serve as potential regulators of breast epithelial differentiation. Previous studies have found that miRNA signatures of purified breast cancer stem cells (BCSCs) and bulk population differ in both normal and malignant breast tissues [3–7]. miR-200 family members are significantly downregulated in both BCSCs and MaSCs, and miR-200c over-expression can reduce tumor initiation of BCSCs and suppress mammary duct formation by MaSCs [4]. Let-7 and miR-93 act in similar fashion in BCSCs [5, 6]. miR-221 has been shown to interfere with the cell cycle in breast cancer [8], but no report about its influence on hierarchy of normal and malignant breast epithelium has been published. In this study, we examined miR-221 expression in different hierarchical subpopulations from normal and malignant breast epithelium, and demonstrated the unique properties of miR-221 in regulating their percentage ratio, which provided important insight into the regulation of miR-221 on normal and malignant breast epithelial cells.

## RESULTS

### miR-221 expression varies in different subpopulations of normal human breast epithelium

**Figure 1:**
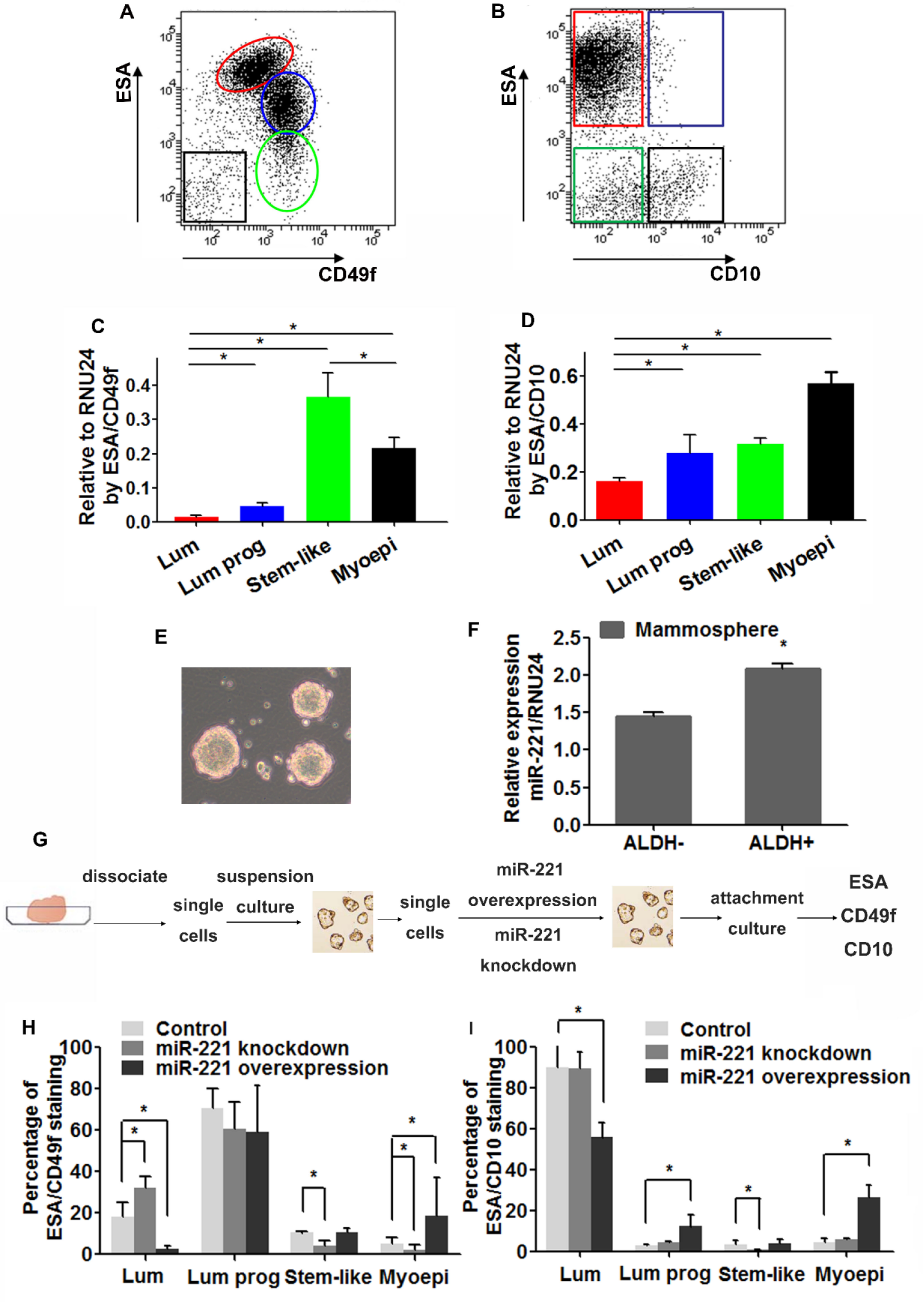
Expression of miR-221 and its role in hiercharies of human mammary epithelium **(A, B)** Single cells separated from reduction mammoplasty were immunostained with ESA, CD49f, and CD10 antibodies, and analyzed by FACS. Hierarchical subpopulations were indicated by colored circles or boxes (Red color, ESA+CD49f^low^/ESA+CD10-: luminal cells; Blue color, ESA^+^CD49f^ high^/ESA^+^CD10^+^: luminal progenitor cells; Green color, ESA^−^CD49f^ high^/ESA^−^CD10^−^: stem-like cells; Black color, ESA^−^CD49f^−^/ESA^−^CD10^+^: myoepithelial cells). **(C, D)** Expression level of miR-221 was assessed by qRT-PCR in subpopulations separated by FACS. Relative miRNA expression was normalized to RNU24. Data represent means ± SEM of 3 wells. (*p < 0.05). **(E)** Mammospheres were generated from Single mammary cells in serum free medium. **(F)** Mammospheres were dissociated into single cells and sorted into ALDH− and ALDH+ cells by ALDEFLUOR assay using FACS. miR-221 levels in both populations were examined by qRT-PCR. Relative miRNA expression was normalized to RNU24. Data represent means ± SEM of 3 wells. (*p < 0.05). **(G)** Schematic of experimental design: single cells dissociated from mammospheres were infected with lentiviruses to induce or knock-down miR-22, and cultivated in suspension to enrich stem-like cells and plated in dishes with collagen substratum to induce differentiation, and immunostained for ESA, CD49f, and CD10. **(H, I)** Percentages of differentiated subpopulations after induction and knockdown of miR-221 in normal breast stem-like cells. Cells were stained by ESA/CD49f (H) and ESA/CD10 (I) and analyzed by FACS. Data represent means ± SEM, (*p < 0.05, *n* = 3).

Subpopulations of human mammary epithelium can be stained with differentiation related cell surface markers: epithelial surface antigen (ESA) [9, 10], epithelial progenitors α6-integrin (CD49f) [2], and common acute lymphoblastic leukemia antigen (CD10) [11]. After depleting hematopoietic, endothelial and mature red blood cells by fluorescence-activated cell sorting (FACS) [12, 13], epithelial cells from normal breast reduction mammoplasty were separated into four subpopulations using two marker sets (ESA/CD49f and ESA/CD10): stem-like cells (ESA^−^CD49f^+^, ESA^−^CD10^−^), luminal progenitor cells (ESA^+^CD49f^+^, ESA^+^CD10^+^), mature luminal cells (ESA^+^CD49f^−^, ESA^+^CD10^−^), and stromal/myoepithelial cells (ESA^−^CD49f^−^, ESA^−^CD10^+^) (Figure 1A and 1B) [2,3,14]. Quantitative reverse transcriptase polymerase chain reaction (qRT-PCR) analysis on these populations revealed that miR-221 expression was higher in myoepithelial and luminal progenitor cells than in mature luminal cells, by both staining sets (Figure 1C and 1D). And by ESA/CD49f alone (a more commonly used staining set), miR-221 was also higher in stem-like cells (Figure 1C).

To further examine this expression pattern, cells from normal breast reduction mammoplasties were cultured in serum-free medium to generate mammospheres (Figure 1E). The Aldehyde Dehydrogenase (ALDH) positive stem-like cells from the mammospheres, which are capable of self-renewal and multi-lineage differentiation [15], showed a significantly higher miR-221 expression level compared to ALDH^−^ cells, as assessed by qRT-PCR (Figure 1F). These results suggest that in normal breast tissue, elevated miR-221 expression is more common in higher-ranked hierarchical subpopulations and myoepithelial cells.

### miR-221 is sufficient to regulate hierarchy during differentiation of normal breast stem cells

Owing to the distinct expression pattern of miR-221 in normal mammary lineage subpopulations, we asked whether modulation of miR-221 levels would change the proportion of cell lineages during MaSC differentiation. We used a doxycycline (Dox)-inducible lentiviral miR-221 construct tagged with RFP (pTRIPZ-mir-221-RFP) and mirZip anti-sense miRNA (mirZip221-DsRed) to determine the functional role of miR-221. Cells from mammospheres were dissociated into single cells and transduced with lentiviruses. Transduced cells were cultured for ten days in serum-free medium to generate mammospheres, which were dissociated into single cells again and cultured on collagen substratum in serum-containing medium to induce differentiation (Figure 1G) [12, 15]. After ten days, by both FACS staining sets (ESA/CD49f and ESA/CD10), induction of miR-221 in MaSCs generated more myoepithelial cells and less luminal cells during differentiation. By ESA/CD49f alone, knockdown of miR-221 in MaSCs resulted in more luminal cells and less myoepithelial cells (Figure 1H and 1I). These results suggest that the increased miR-221 level in MaSCs promoted myoepithelial differentiation, whereas miR-221 downregulation favored luminal differentiation.

**Figure 2:**
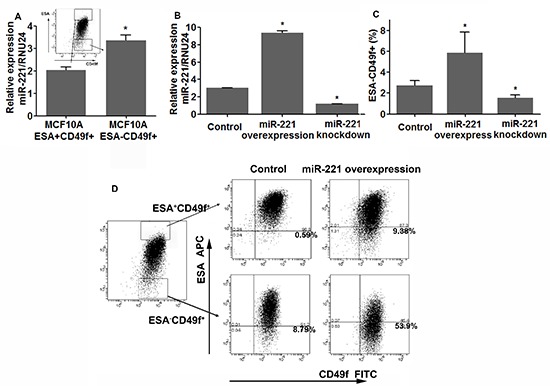
miR-221 regulates the percentage of stem-like cells and induces EMT in immortalized normal breast cell line MCF10A **(A)** ESA^+^CD49f^+^ and ESA^−^CD49f^+^ MCF10A cells were sorted by FACS, and expression levels of miR-221 in both populations were assessed by qRT-PCR. Relative miRNA expression was normalized to RNU24. Data represent means ± SEM of 3 wells. (*p < 0.05). **(B)** miR-221 over-expression and repression were confirmed by qRT-PCR in MCF10A cells after being transfected with pTRIPZ and MirZIP lentivirus. **(C)** The percentage of ESA-/CD49f+ subpopulation was analyzed by FACS after 3-day induction and knockdown of miR-221 in MCF10A cells. Data represent means ± SEM, (*p < 0.05, *n* = 3). **(D)** ESA+CD49f + and ESA−CD49f + MCF10A cells were sorted using FACS, and miR-221 was induced in both populations for 5 days, and ESA/CD49f staining was analyzed again by FACS 5 days later.

To validate our results, we used the non-tumorigenic human breast epithelial cell line MCF10A [16]. The MCF10A cells transcriptionally and functionally resembles luminal progenitor cells [13, 15, 17], and display two phenotypes [luminal-like (ESA^+^CD49f^+^) and basal-like (ESA^−^CD49f^+^)] which can mutually switch under certain condition both *in vitro* and *in vivo* [18, 19]. Under normal culture conditions, most MCF10A cells exhibit luminal-like (ESA^+^CD49f^+^) phenotype with a small amount of cells under basal-like phenotype (Figure 2A). Both cell populations were isolated by FACS, and qRT-PCR analysis showed that miR-221 expression was significantly higher in the basal-like population (ESA^−^CD49f ^ +^) as compared to the luminal-like population (ESA^+^CD49f  ^+^) (Figure 2A). Then we over-expressed and knocked down miR-221 in MCF10A (Figure 2B) using the same lentiviral systems as previously described. FACS analysis showed that, induction of miR-221 significantly expanded basal-like cells (ESA^−^CD49f^+^), whereas knock-down of miR-221 suppressed basal-like cells and enriched luminal-like cells (Figure 2C). To find the source of expanded basal-like cells by miR-221 overexpression, ESA^−^CD49f^+^ and ESA^+^CD49f^+^ cells were cultivated with or without induction of miR-221 for five days (Figure 2D). Under normal culture conditions, basal-like cells (ESA^−^CD49f^+^) can generate luminal-like cells (ESA^+^CD49f^+^) exhibiting bi-potent potential [18, 19], whereas luminal-like cells rarely generate basal-like cells. However, after 5 days of miR-221 induction, almost half basal-like cells retained their phenotype and luminal-like cells became capable of forming basal-like cells (Figure 2D). Taken together, miR-221 is sufficient to regulate the cell differentiation where miR-221 over-expression favored myoepithelial type differentiation and miR-221 inhibition promotes luminal type differentiation.

### miR-221 expression varies in different breast cancer subtypes and is correlated with clinical outcome

**Figure 3:**
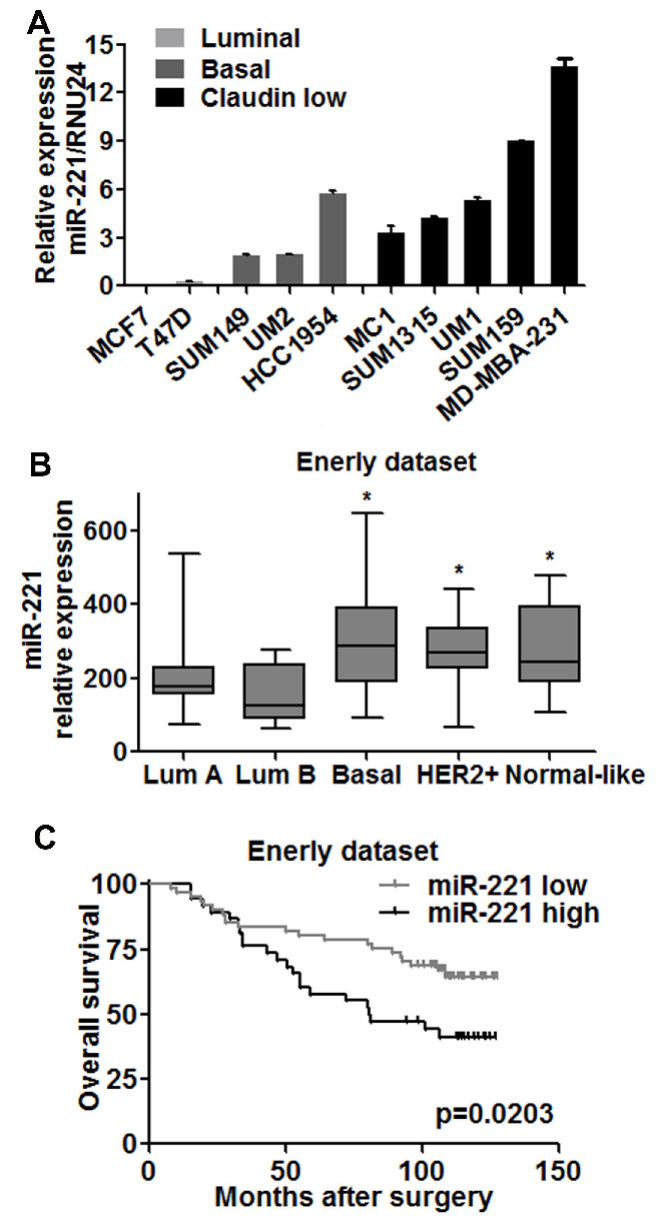
miR-221 is upregulated in less differentiated breast cancer cells and correlated with clinical outcome **(A)** Expressions of miR-221 in luminal (grey), basal (dark) and claudin-low (black) types of breast cancer cell lines were assessed by qRT-PCR analysis. Relative miRNA expression was normalized to RNU24. Data represent means ± SEM of 3 wells. (*p < 0.05). **(B)** Based on Enerly dataset, miR-221 levels in Basal (*n* = 15), Her2 over-expression (*n* = 17) and Normal-like (*n* = 9) subtypes of breast cancer were higher than that in luminal A (*n* = 39) and B (*n* = 12) types of cancer respectively. Student T test was applied, (*p < 0.05). **(C)** miR-221 expression was negatively correlated with overall survival of breast cancer patients based on Enerly (*n* = 99) dataset by the log-rank test (*p* = 0.0203). Patients were divided into high and low groups by median value of miR-221.

Given the linkage between normal breast cell subpopulations and tumor subtypes [2, 3], we examined miR-221 expression in different subtypes of breast cancer cells: luminal (T47D, MCF7), basal (SUM149, HCC1954), and Claudin-low (SUM159, SUM1315, MDA-MB-231) and established primary breast cancer xenografts in non-obese diabetic/severe combined immunodeficiency (NOD/SCID) mice [MC1 (Claudin-low), UM1 (Claudin-low) and UM2 (Basal)] [6]. qRT-PCR analysis revealed that miR-221 was barely detected in luminal type, and highly expressed in basal and Claudin-low type of breast cancer (Figure 3A).

Since cancer cell lines and tumor xenografts may not biologically recapitulate the primary tumor, we investigated miR-221 levels in a publicly available breast cancer microarray dataset (Enerly dataset [20]). In this dataset, the miR-221 level was upregulated in basal, Her2^+^ and Claudin-low subtypes compared to luminal A and luminal B tumors respectively (Figure 3B). Notably, patients with higher miR-221 levels had poor overall survival (Figure 3C). Taken together, these results suggest that miR-221 is correlated with the differentiation of breast cancer and clinical outcomes.

### miR-221 promotes stem-like properties in luminal type of breast cancer

**Figure 4:**
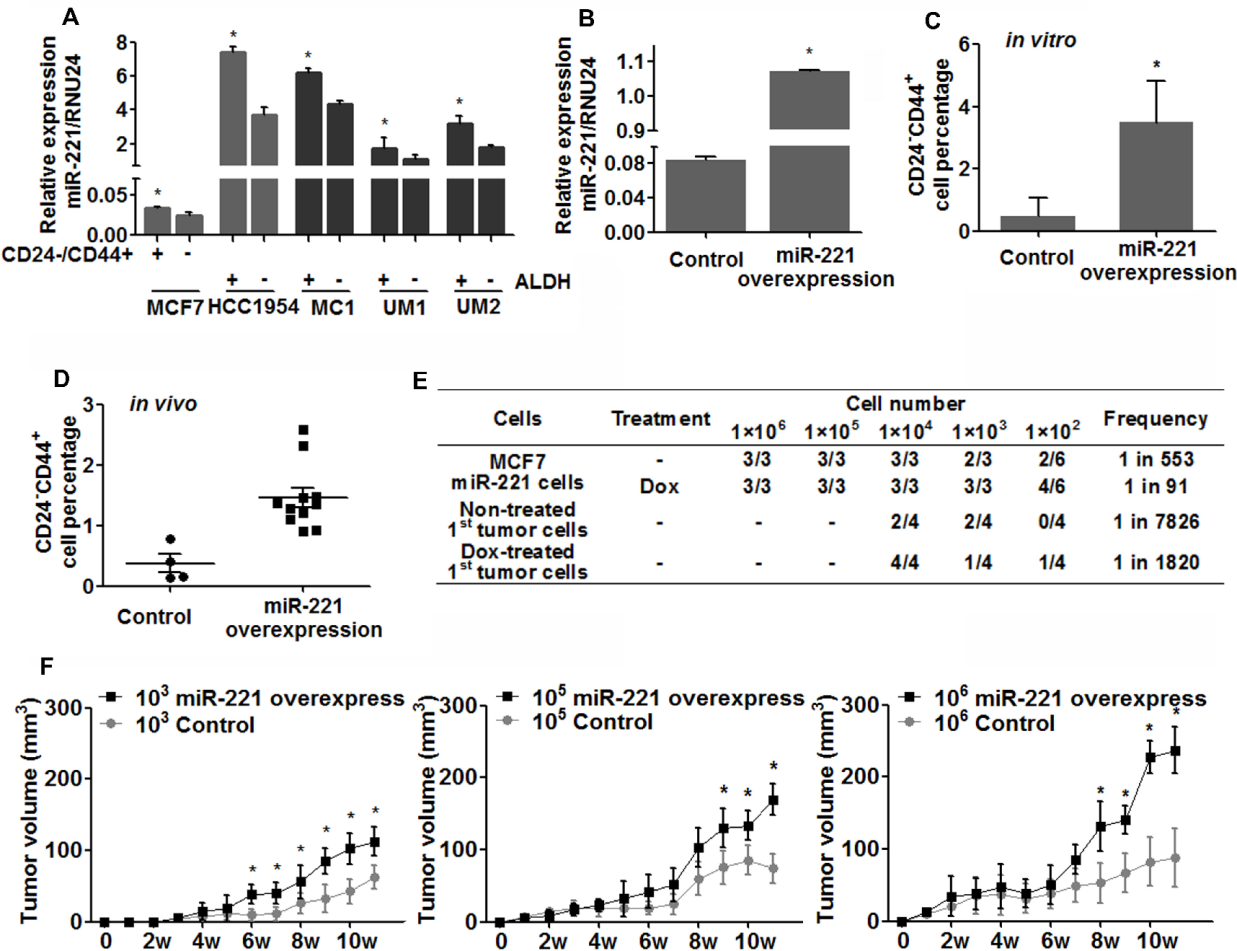
Expression of miR-221 and its role in regulating breast cancer stem-like cells **(A)** Stem-like cells and bulk cells were isolated from breast cancer cell lines (grey) and primary human breast tumor xenografts (black) by ALDH or CD24/CD44 staining, and miR-221 expression was assessed by qRT-PCR. Relative miRNA expression was normalized to RNU24. Data represent means ± SEM of 3 wells. (*p < 0.05). **(B)** miR-221 over-expression was confirmed by qRT-PCR in MCF7 cells after being transfected with pTRIPZ lentivirus and treated with DOX (1 μg/ml) for 3 days. **(C)** Stem-like cells (CD24^−^CD44^+^) was enriched after over-expression of miR-221 in MCF7 cells *in vitro*. Data represent means ± SEM, (*p < 0.05), *n* = 3. **(D)** pTRIPZ-hsa-miR-221 lentivirus transduced MCF7 cells were implanted into NOD/SCID mice at serial dilutions (10^3^, 10^5^, and 10^6^ cells per mice), and DOX was added into the drinking water (1 mg/ml) to induce miR-221 throughout the experimental period. At 12 weeks, mice were sacrificed and tumor cells were harvested and dissociated into singe cells. Percentage of stem-like MCF7 cells (CD24−CD44+) were assessed by FACS analysis (D) Data represent means ± SEM, (*p < 0.05). **(E)** Tumor initiating frequency was examined in 1^st^ and 2^nd^ generation of tumors. MCF7 cells isolated from 1^st^ generation of tumors were planted into secondary NOD/SCID mice with no further miR-221 induction (no DOX water). Tumor initiating frequency was calculated via the L-Calc Software [1, 32]. **(F)** Sizes of tumors were monitored weekly. The “*” in the tumor growth curve indicates that the tumor growth is significantly different between the control group.

Because miR-221 was able to affect MaSCs differentiation, we wondered miR-221 might also regulate BCSCs. Using proven BCSCs markers (ALDH or CD24^−^CD44^+^) [21, 22], qRT-PCR analysis revealed that miR-221 level was higher in stem-like cells than in bulk cells from both luminal and basal type of breast cancer cell lines, regardless of tumor subtype (Figure 4A). To determine the effect of miR-221 on BCSCs, we next induced miR-221 in the luminal cell line MCF7, which express a very low basal miR-221 level (Figure 4B). FACS analysis found the stem-like cell population (CD24^−^CD44^+^) was significantly expanded by over-expression of miR-221 *in vitro* (Figure 4C). To validate this result *in vivo*, miR-221 overexpressing MCF7 cells were implanted into the mammary fatpads of NOD/SCID mice at serial dilutions (10^3^, 10^5^, 10^6^) to determine the tumor initiation ability. In agreement with the *in vitro* results, CD24^−^CD44^+^ population within tumors was expanded after twelve weeks of miR-221 over-expression (Figure 4D), and tumor-initiating frequency increased from 1 in 533 cells to 1 in 91 cells (5.86 times) (Figure 4E).

To further determine whether miR-221 could promote CSC self-renewal, we implanted MCF7 cells isolated from 1^st^ generation of tumors into secondary NOD/SCID mice [21, 22], with no further miR-221 stimulation (no doxycycline). Tumor initiation in 2^ndary^ transplantation was significantly higher in mice implanted with miR-221 over-expressed cells than control cells (1 in 1820 cells vs 1 in 7826 cells) (Figure 4E). In addition, tumor growth was also promoted compared to control group (Figure 4F). We also induced miR-221 in claudin-low and basal type of breast cancer cells (HCC1954, SUM149, SUM159), which possess high basal miR-221 level. No difference in the stem-like populations was found after induction of miR-221, whereas apoptosis was rapidly observed after miR-221 knock-down in these cell lines (data not shown). Taken together, these results suggested that miR-221 was able to stimulate stem-like properties in breast cancer cells, especially in the luminal type of breast cancer.

### The EMT related gene ATXN1 is the target of miR-221 in regulating normal and malignant breast stem-like cells

**Figure 5:**
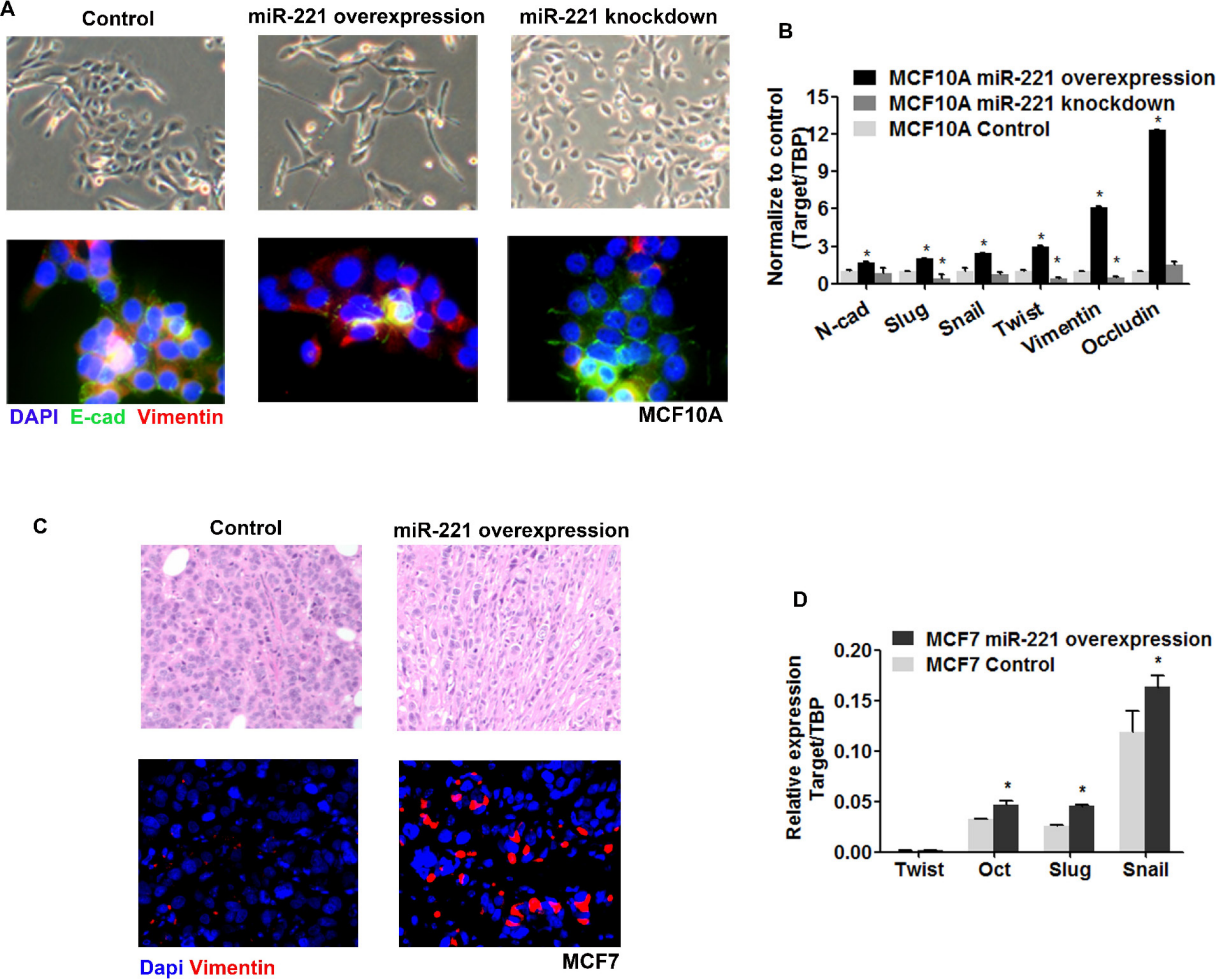
EMT participate in the regulation of miR-221 in normal and malignant breast cells **(A)** Epithelial and mesenchymal morphologies were observed after miR-221 over-expression and inhibition (upper line), and was confirmed by immunofluorescence staining of E-cadherin and Vimentin (bottom line). Cells were counterstained with DAPI. **(B)** EMT related genes (N-cadherin, Slug, Snail, Twist, Vimentin, and Occludin) were examined after over-expression and knock-down of miR-221 in MCF10A cells by qRT-PCR. Relative mRNA expression was normalized to TBP. Data represent means ± SEM of 3 wells. (*p < 0.05). **(C)** Mesenchymal morphology was induced by overexpression of miR-221 in MCF7 tumors, as observed by H&E staining (upper line). Vimentin-positive cells (Red) were stimulated by overexpression of miR-221 in MCF7 tumors, as observed by immunofluorescence staining. Cells were counterstained with DAPI (bottom line). **(D)** EMT related genes (Twist, Oct, Slug, and Snail) were examined after over-expression of miR-221 in MCF7 tumors as assessed by qRT-PCR.

EMT participates in mammary tissue development and differentiation, and can generate cells with properties of stem cells. We asked whether EMT was involved in the phenotypical changes induced by miR-221. After induction of miR-221 in MCF10A cells, spindle fibroblast-like cells were observed, while smaller-sized round-shape morphology was induced after miR-221 knock-down (Figure 5A, upper line). One of the hallmarks of EMT is the loss of membrane E-cadherin and concomitant expression of Vimentin. Using immunofluorescence staining, induction of miR-221 inhibited E-cadherin and stimulated Vimentin expression in MCF10A cells, whereas miR-221 inhibition decreased Vimentin expression and re-established the membrane distribution of E-Cadherin (Figure 5A, bottom line). Additionally, EMT related genes (N-cadherin, Slug, Snail, Twist, Vimentin, and Occludin) were upregulated by induction of miR-221, and downregulated by miR-221 knock-down (Figure 5B). Similarly, a mesenchymal morphology was observed in miR-221 induced MCF7 tumors (Figure 5C, upper line). miR-221 over-expression stimulated Vimentin in MCF7 tumors by immunofluorescence staining (Figure 5C, bottom line). And some EMT genes were also upregulated (Twist, Oct, Slug, Snail) in miR-221 induced MCF7 tumors (Figure 5D). Combining these results, we considered EMT was involved in the regulation by miR-221 in normal and malignant breast tissue.

**Figure 6:**
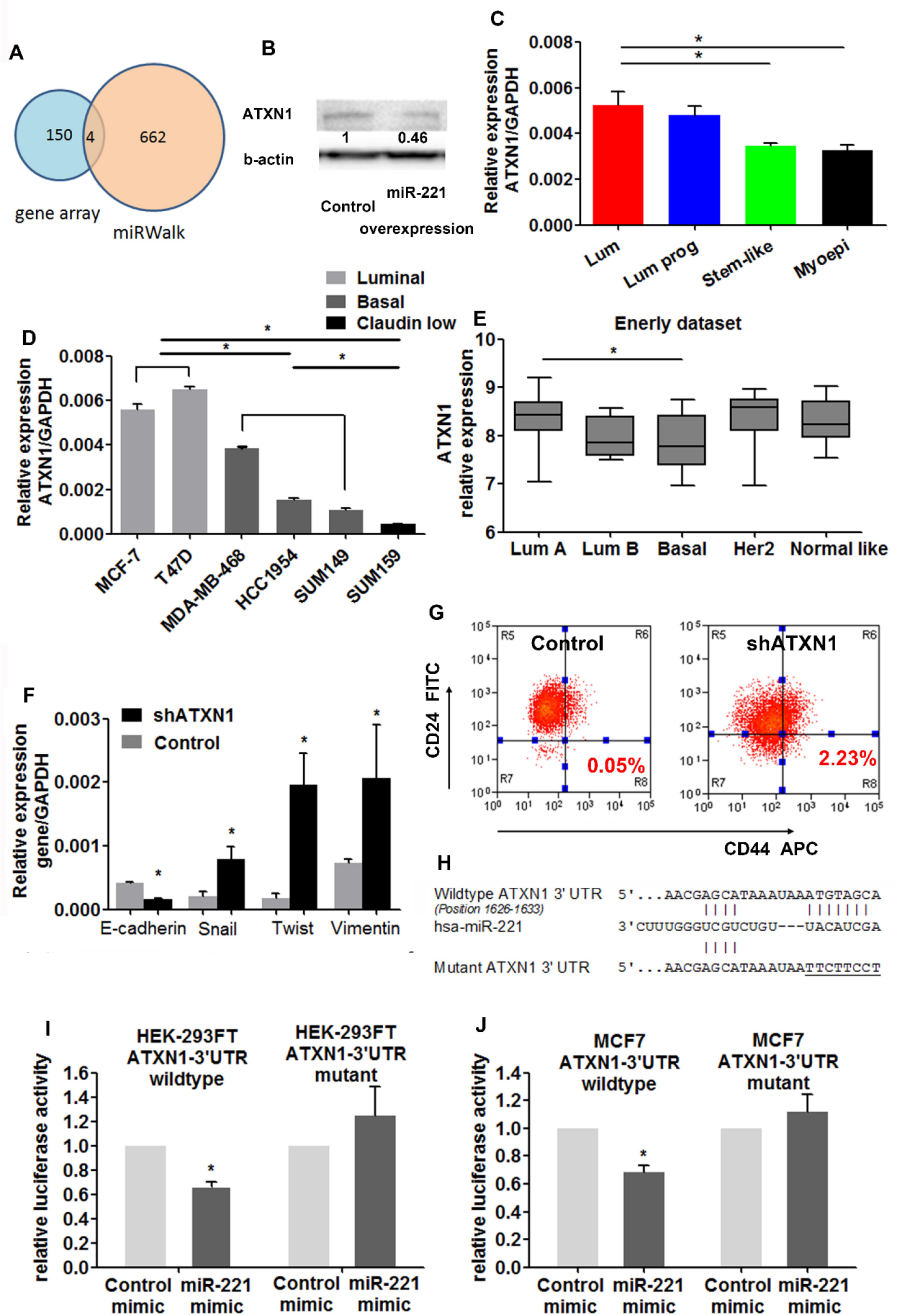
miR-221 targets ATXN1 in normal and malignant breast stem-like cells **(A)** By mRNA profiling, 150 genes were downregulated by overexpression of miR-221. 662 genes were potential targets of miR-221 predicted by miRWALK. Four candidate genes (ATXN1, FNDC3A, ERBB3, PSD3) were overlapped. **(B)** Western blot analysis showed that overexpression of miR-221 downregulated ATXN1 protein level in MCF7 cells. **(C)** qRT-PCR showed that ATXN1 expression levels were higher in luminal breast cells, and lower in myoepithelial cells sorted from normal mammary tissue by ESA/CD10 staining. Data represent means ± SEM, (*p < 0.05, *n* = 3). **(D)** Expressions of ATXN1 in luminal (grey), basal (dark) and claudin-low (black) types of breast cancer cell lines were assessed by qRT-PCR analysis. Relative mRNA expression was normalized to GAPDH. Data represent means ± SEM of 3 wells. (*p < 0.05). **(E)** Transcriptional array level of ATXN1 in Enerly dataset was higher in luminal A type of breast cancer (*n* = 41) than in basal type of tumors (*n* = 15, *p* = 0.0044). **(F)** mRNA level of EMT related genes (E-cadherin, Snail, Twist, Vimentin) were examined after knocking down ATXN1 in MCF7 cells. Relative mRNA expression was normalized to GAPDH. Data represent means ± SEM of 3 wells. (*p < 0.05). **(G)** Percentage of stem-like cells (CD24-CD44+) was examined after knocking down ATXN1 in MCF7 cells by shRNA lentivirus. Data represent means ± SEM, (*p < 0.05, *n* = 3). **(H)** Sequence alignment of miR-221 and the target site (wild-type and mutant) in 3′ UTRs of ATXN1. **(I, J)** Reporter assay in HEK-293FT (I) and MCF7 cells (J), with co-transfection of Wt or mut-reporter and control-miR, or miR-221 mimic as indicated. Each bar represents values from 3 independent experiments. (*p < 0.05).

To identify candidate targets of miR-221, we used genome-wide mRNA expression profiling data obtained from miR-221 induced and control MCF10A cells. One hundred and fifty genes were downregulated by overexpression of miR-221 (Supplementary Table 1). To identify the direct targets of miR-221, these results were compared with potential miR-221 targets predicted by miRWALK, a program combining ten miRNA target prediction databases [23]. Four candidate target genes (ATXN1, FNDC3A, ERBB3, PSD3) were overlapped (Figure 6A). ATXN1 (Ataxin-1), a polyglutamine protein that alters cell morphology by interacting with microtubules during neuronal development [24, 25], drew particular attention. ATXN1 was found to occupy the promoter region of E-cadherin gene and hence activate its expression [24]. ATXN1 showed opposite mRNA patterns against miR-221 in normal breast cell subpopulations (Figure 6C), breast cancer cell lines (Figure 6D) and breast cancer subtypes from the Enerly dataset (Figure 6E). MCF7 cell line was chosen for further experiments owing to the low basal level of miR-221 (Figure 3A) and high level of ATXN1 (Figure 6B). Induction of miR-221 led to a decrease of ATXN1 protein in MCF7 cell line by western blot (Figure 6B). To examine whether ATXN1 inhibition would mimic the effect of miR-221 overexpression, the ATXN1 gene was knocked down in MCF7 cells by short hairpin RNA (shRNA). Expression levels of Snail, Twist, Vimentin were upregulated, E-cadherin was downregulated as assessed by qRT-PCR (Figure 6F). FACS analysis revealed that stem-like cells (CD24^−^CD44^+^) expanded after ATXN1 knockdown (Figure 6G), resembling the effect of miR-221 overexpression.

To confirm ATXN1 was directly targeted by miR-221, a luciferase reporter assay was utilized combined with site-directed mutagenesis. 3′-untranslated regions (3′-UTRs) of wild-type and mutated ATXN1 gene (Figure 6H) was cloned at downstream of Renilla luciferase in a dual-luciferase reporter vector and co-transfected with control or miR-221 mimics into HEK-293FT cells. Relative luciferase activity for wild-type ATXN1 was reduced after adding miR-221 mimics (Figure 6I), whereas mutations in predicted target site of 3′-UTR of ATXN1 gene abrogated the inhibition by miR-221 mimic, indicating that ATXN1 gene is the direct target of miR-221. This result was repeated in MCF7 cell line (Figure 6J), showing that the observed effects were not restricted to one cell line. Together, these results suggest that ATXN1 may be the direct target of miR-221 in regulating breast stem-like cells.

## DISCUSSION

Within human normal mammary tissue, epithelial stem cells undergo differentiation and ultimately generate two mature cell lineages: luminal and myoepithelial cells. miRNA profiling on the parental and offspring breast cells showed dramatic differences [4, 7], suggesting that miRNAs might participate in the differentiation process. Indeed, certain miRNAs such as miR-200 and miR-93 has been found to regulate MaSC and BCSC differentiation [4, 6]. Here we present data examining miR-221 expression in the hierarchy of normal and malignant breast cells and demonstrate the properties of miR-221 in regulating hierarchies in normal and malignant breast tissue.

One of the key findings of this study is that miR-221 participates in the self-balance of mammary hierarchical subpopulations. miR-221 was up-regulated in normal stem-like and myoepithelial cells, and decreased in mature luminal cells. Furthermore, during stem cell differentiation, over-expression of miR-221 resulted in more myoepithelial cells and knock-down of miR-221 gave rise to more luminal cells. We used two sets of cell surface markers for cell lineage separation because there are no staining strategies perfectly discriminating hierarchical subpopulations. Bachelard-Cascales et al [26] found ESA^+^CD10^−^ cells include ESA^+^CD49f^−^ and ESA^+^CD49f^+^ cells, and ESA^+^CD10^+^ cells contain ESA^+^CD49f ^−^ cells, thus ESA/CD10 stains a wider hierarchical range than ESA/CD49f and make the results more complementary. This could explain why miR-221 levels in luminal and luminal progenitor cells by ESA/CD10 were higher than that by ESA/CD49f staining (Figure 1C and 1D). The results in normal breast cells were in accordance with MCF10A cell line. MCF10A is a phenotypically flexible normal breast cell line representing functional and transcriptional features of both basal progenitor and luminal cells [13, 18]. Likewise, miR-221 over-expression formed more mesenchymal MCF10A cells and miR-221 repression favored luminal-like phenotype. This might be explained by the opposite expression pattern of estrogen receptor against miR-221 in these subpopulations and the negative regulatory loop between estrogen receptor and miR-221 [27]. Given the importance of estrogen receptor in mammary gland development, we propose miR-221 is involved in regulating the differentiation of normal breast epithelial cells.

We also found miR-221 was differentially expressed in subtypes of breast cancer and correlated with clinical outcome. Based on the data from breast cancer cell lines and a large public dataset, we found miR-221 level was higher in claudin-low and basal type of breast cancer than in luminal type of cancer, which is similar to its pattern in normal breast stem-like, myoepithelial and luminal cells. This correlation fits well with the signature similarity between normal and malignant breast hierarchies [2, 3]. Moreover, patients with higher miR-221 levels showed worse overall survival. This can be explained by our findings that, miR-221 over-expression activated EMT and stem-like cells in luminal type of cancer both *in vitro* and *in vivo*. We did not observe more stem-like cells in basal and claudin-low cell lines (HCC1954, SUM149, SUM159) after over-expression of miR-221 (data not shown), probably owing to the high basal levels of miR-221 in these cell lines. Knock-down of miR-221 in these cell lines resulted in rapid apoptosis (data not shown), resembling the findings in hepatocellular cancer and glioblastoma, which also express high basal levels of miR-221 [28, 29]. However, knock-down of miR-221 in primary normal breast cells and MCF10A cells did not induce apoptosis. This is probably due to that primary normal breast cells and MCF10A cells possess more than one cell phenotype, so, the loss of myoepithelial/basal-like cells by miR-221 knock-down can be compensated by the expansion of luminal cells induced.

EMT and the converse process of mesenchymal-to-epithelial transition (MET) play essential roles during mammary tissue development. EMT induced cell plasticity participate in the invasion of the terminal end buds (TEBs) as expanding mammary ducts penetrate into the surrounding stroma. We found typical EMT morphology and activation of EMT-related genes after induction of miR-221 in MCF10A and MCF7 cells *in vitro* and *in vivo*. Using public miRNA datasets, miRNA profiling and luciferase reporter assay, we found the EMT-related gene, ATXN1, was the target of miR-221 in the phenotypical regulation of breast cells. ATXN1 can occupy the promoter region of E-cadherin and activate its transcription [24, 25]. Knock-down of ATXN1 mimicked induction of EMT and expansion of stem-like cells by induction of miR-221 in MCF7 cells. This is one of the few reports about ATXN1's role in breast cancer.

Here, we have uncovered a not previously scrutinized capacity of miR-221 for sustaining breast cell hierarchy in normal and malignant breast tissue, probably via EMT. Combined with the other miRNAs capable of regulating hierarchy in normal and malignant breast tissue (mir-93, mir-200, and let7), we propose that miRNA therapy could be an effective strategy for targeting breast cancer.

## METHODS

### Cell culture

Normal and malignant breast cell lines MCF10A, MCF7, T47D, HCC1954, MDA-MB-468 and MDA-MB-231 were originally obtained from the American Type Culture Collection (ATCC). SUM149, SUM1315 and SUM159 were provided by Dr. Stephen Ethier. Cell lines were cultured at 37°C in a humidified 5% CO2 environment with different medium as previously described [30].

### Lentiviral infection

A highly efficient doxycycline (Dox)-inducible retroviral system (TRIPZ lentivral vector; http://www.openbiosystems.com/RNAi) was used to induce mir-221 over-expressing, mirZIP-lentivector (SBI, Mountain View, CA) was used to generate mir-221-knockdown lentiviruses, and retroviral RFP vector (OriGene, Rockville, MD) was used to generate ATXN1 shRNA virus in UM Vector Core Facility. The cell lines were infected with the lentiviruses as described previously [31].

### Dissociation of mammary tissue and differentiation culture conditions

A total of 100–200 g of normal breast tissue from reduction mammoplasties was minced with scalpels and dissociated enzymatically, and single cells were cultured in suspension to produce mammospheres as previously described [21, 30]. After ten days, the cultures were dissociated into single cells, transduced with lentiviruses, maintained in suspension culture for another seven to ten days, and dissociated again into single cells. Infected cells were plated on collagen-coated plates at a density of 5,000 viable cells per 10-cm-diameter dish. Cells were grown in Ham's F-12 medium (Gibco) with 5% FBS, 5 μg/ml insulin, 1 μg/ml hydrocortisone, 10 μg/ml cholera toxin (Sigma–Aldrich), 10 ng/ml epidermal growth factor (BD Biosciences) and 1X Pen/Strep/Fungizone Mix (Gibco). Cells were collected for the lineage analysis by FACS seven to ten days later. All experiments were conducted in triplicate by using single-cell suspensions derived from three different patients.

### qRT-PCR for miRNA and mRNA

Total RNA for the miRNA and mRNA experiment were extracted from the above cells using a Qiagen miRNeasy Kit (217004) and RNeasy Kit (74104) (Valencia, CA, USA), respectively. qRT-PCR was performed using a TaqMan MicroRNA Reverse Transcription Kit and/or TaqMan PCR Kit (Applied Biosystems, Foster City, CA, USA) and the Applied Biosystems 7900HT Sequence Detection System (Applied Biosystems). The appropriate TaqMan probes were purchased from Applied Biosystems for qRT-PCR quantification of miR-221, E-cadherin, N-cadherin, Vimentin, Twist, Snail, Slug, and Oct. PCR reactions for each sample were run in triplicate. The number of cycles required for amplification to reach the threshold limit, the Ct-value was used for quantification. RNU24 was used as an endogenous control for miRNA data normalization, and TBP was used as an endogenous control for other gene normalization. All TaqMan miRNA assays used in this study were obtained from Applied Biosystems.

### Flow cytometry analysis and sorting

Flow cytometry was performed using an Astrios flow cytometer (Beckman coulter, Inc. Brea, CA, USA). Conjugated mouse anti-human antibodies APC anti-ESA, APC anti-CD49f, APC anti-CD44, FITC anti-CD24, PE anti-CD45, PE anti-CD31, PE anti-CD235 and anti-mouse H2KD were purchased from BD Pharmingen (San Diego, CA, USA). Unconjugated mouse anti-human antibody CD10 (BD Pharmingen) was used with FITC conjugated donkey anti-mouse secondary antibodies (Jackson ImmunoResearch Laboratories, West Grove, PA). Cells were harvested by dissociation using 0.05% trypsin/EDTA. 1 × 106 cells were resuspended in 200 μl HBSS with 2% FBS and then stained with the proper amount of antibodies (according to the instruction sheet) for 30 minutes at 4°C. Cells incubated with unconjugated antibodies were stained with secondary antibodies for another 30 min at 4°C. For the Aldefluor assay, the ALDEFLUOR kit (StemCell Technologies Inc., Vancouver, BC, Canada) was used and perform as previously described [21]. Dead cells were excluded by DAPI staining (0.2 μg/ml). Stem cell percentages in tumor cells were analyzed after gating out H2KD positive mouse cells.

### Breast cancer xenograft assay in NOD/SCID mice

All mice were housed in the AAALAC-accredited specific pathogen-free rodent facilities at the University of Michigan. Mice were housed on sterilized, ventilated racks and supplied with commercial chow and sterile water that had been previously autoclaved. All experimentation involving live mice were conducted in accordance with standard operating procedures approved by the Committee on the Use and Care of Animals at the University of Michigan. Six-week-old female NOD/SCID mice were purchased from Jackson Laboratories (Strain #1303) (Bar Harbor, ME), and 103, 105, and 106 pTRIPZ-hsa-miR-221 MCF7 cells were injected into their mammary fat pads. The mice were then treated with and without doxycycline (DOX, 1 mg/ml in drinking water) after surgery. Tumor size was measured twice a week. The animals were euthanized when the tumors were approximately 1–1.5 cm at their largest diameter. Part of each tumor was then formalin fixed and paraffin embedded for histology and tumor fragments were enzymatically digested and filtered to generate single cells for CD44/CD24 FACS staining. Secondary tumor formation was assessed by injection into female NOD/SCID mice with 10^2^, 10^3^, 10^4^ cells from DOX-treated and untreated tumors, in absence of DOX water afterwards. Tumor initiating frequency was calculated by limiting dilution analysis [21] and the L-Calc Software [32] (StemCell Technologies Inc., Vancouver, BC, Canada). When assessing the miR-221 level in breast cancer cells, human primary breast cancer xenografts MC1, UM1 and UM2 were used in addition to the established cell lines, as previously described [6].

### Immunostaining

For staining, paraffin-embedded sections of xenografts were deparaffinized in xylene and rehydrated in graded alcohol. Antigen enhancement was conducted by incubating the sections in citrate buffer at pH 6.0 (Dakocytomation, Copenhagen, Denmark) according to the manufacturer's recommendations. Vimentin (BD biosciences, San Jose, CA) was applied at a 1:25 dilution and incubated for 1 hour. PE-labeled secondary antibody (Jackson ImmunoResearch Laboratories, West Grove, PA) was used at a 1:250 dilution and incubated for 20 minutes. Nuclei were counterstained with DAPI/antifade (Invitrogen, Carlsbad, CA) and cover-slipped. Sections were examined on Olympus FV-500 Confocal fluorescent microscope.

### Microarray data analysis

Public array dataset: data types, platforms and analyses are as previously described [33]. Normalized miRNA and mRNA profiling data and clinical information were downloaded from the GEO database (GSE19536: Enerly dataset [20]). Samples were cross-referenced by tumor IDs. Patients were divided into high and low groups according to the median value of miR-221. The log-rank test was used for survival analysis. Transcriptional profiling: affymetrix human U133 Plus 2.0 was used. Preparation of the cRNA, hybridizations, washes, and detection were conducted according to the manufacturer's protocols (http://www.affymetrix.com/index.affx). Expression data were analyzed by the Robust Multichip Average (RMA) method in R with Bioconductor and associated packages [33]. miRNA family data and predicted target gene list crossed from 10 miRNA databases were downloaded from the miRWALK website (http://www.umm.uni-heidelberg.de/apps/zmf/mirwalk/, combining DIANAmT, miRanda, miRDB, miRWalk, RNAhybrid, PICTAR4, PICTAR5, PITA, RNA22 and Targetscan) [23]. Target genes shared by more than 5 databases were chosen for further experiments.

### Target *in vitro* assays

For luciferase reporter experiments, the 3′ UTR segments of wild type and mutated ATXN1 gene were amplified by PCR from human genomic DNA and inserted into the XhoI and NotI sites of psiCHECKTM-2 vector (Promega) immediately downstream from the stop codon of Renilla luciferase. HEK-293FT and MCF7 cells were cotransfected by using DharmaFECT Duo Transfection Reagent (Thermo Scientific, Hudson, NH, USA) according to the manufacturer's protocol in 96-well plates with 0.1 μg of the luciferase report vector. For each well, 100 nM miRNA oligonucleotides or scrambled oligonucleotides (GenePharma, Shanghai, China) were used. Firefly and Renilla luciferase activities were measured consecutively by using a dual-luciferase reporter assay system (#E1910, Promega) 24 h after transfection.

### Statistical analysis

Data were analyzed using Excel (Microsoft) and GraphPad Prism (Prism). Results are presented as mean ± SEM of the results of three or more independent experiments. Statistics were performed using one-way ANOVA, Mann Whitney U non-parametric and Student *T* test in GraphPad Prism. Data were considered significant if (*P* < 0.05).

## SUPPLEMENTARY TABLE




