# Supplementary material for: Role of microRNA221 in regulating normal mammary epithelial hierarchy and breast cancer stem-like cells
Source: Oncotarget. 2015 Feb 17;6(6):3709–21. doi: 10.18632/oncotarget.2888 (PMC4414148; doi:10.18632/oncotarget.2888)
Supplement: Supplementary file 1 [file oncotarget-06-3709-s001.pdf]

SUPPLEMENTARY TABLE

Supplementary Table 1: mRNA was extracted after 3-day induction of miR-221 in MCF10A cells. Genes changed over 1 fold were listed
